# Surrey County Hospital

**Published:** 1903-08-15

**Authors:** W. F. Patrick

**Affiliations:** Assistant Secretary of the Royal Surrey County Hospital, Guildford


### EDITOR'S LETTER-BOX.

### SURREY COUNTY HOSPITAL.

MR. W. F. PATRICK, Assistant Secretary of the Royal Surrey County Hospital, Guildford, writes: I notice in your issue of August 8th, 1903, page 338, you have a brief review of this hospital. In the last paragraph, however, you imply that no statement of results is issued, and that the tables given are therefore useless. I enclose a copy of last report, on page 12 of which you will find a statement of results, and shall be obliged if you will kindly correct the impression given in your next issue.

[If Mr. Patrick will kindly read through the notice again, he will see that we give *in extenso* all the information that is in the annual report of the Surrey County Hospital under the heading of results of treatment, and therefore our article can mislead no one. Such a table as occupies six lines in an annual report under the heading of results of treatment conveys no meaning to the public and is perfectly useless for any scientific purpose whatever. We have always maintained that the annual report of every hospital ought to contain an anæsthetic table, a table showing the results of operations, and separate tables of the results of treatment of the various medical cases.—ED. THE HOSPITAL.]

